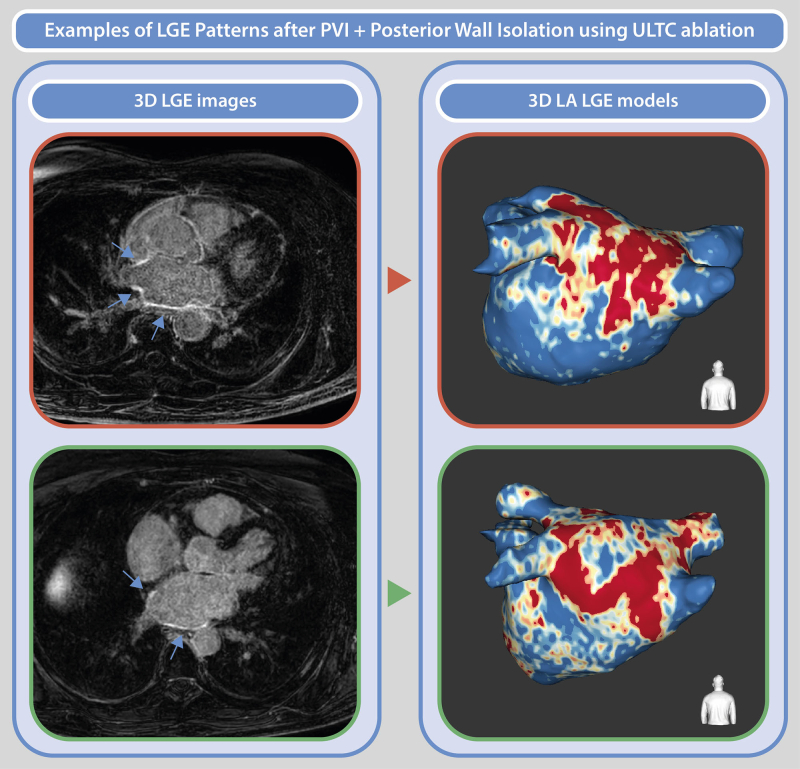# Ablation lesion patterns in atrial fibrillation: A cardiac MRI comparison of different ablation strategies

**DOI:** 10.1016/j.hroo.2026.03.035

**Published:** 2026-04-08

**Authors:** Nikki van Pouderoijen, Marisa van der Graaf, Bob G.S. Abeln, Michiel J.B. Kemme, Max Liebregts, Lucas V.A. Boersma, Cornelis P. Allaart, Luuk H.G.A. Hopman, Marco J.W. Götte

**Affiliations:** 1 Department of Cardiology, Amsterdam UMC, Amsterdam, The Netherlands; 2 Department of Cardiology, St. Antonius Hospital, Nieuwegein, The Netherlands; 3 Department of Cardiology, Radboud University Medical Center, Nijmegen, The Netherlands; 4 Division of Cardiology, Department of Cardiac Sciences, Cumming School of Medicine, Libin Cardiovascular Institute, Stephenson Cardiac Imaging Centre, University of Calgary, Calgary, Canada

**Keywords:** Atrial fibrillation, Pulmonary vein isolation, Cardiac MRI, Late gadolinium enhancement, Lesion characterization, Pulsed field ablation

## Abstract

**Background:**

Pulmonary vein (PV) isolation can be performed using various ablation strategies, including point-by-point radiofrequency (RF) and single-shot ablation techniques. However, the resulting lesion characteristics of these approaches are not yet fully understood. Late gadolinium enhancement cardiac magnetic resonance (LGE-CMR) provides a noninvasive means to assess atrial ablation lesions.

**Objective:**

This study aimed to characterize ablation modality-specific left atrial LGE-CMR lesion patterns.

**Methods:**

Ablation lesion patterns of 42 patients were evaluated on LGE-CMR acquired 3 months after PV isolation was performed with point-by-point moderate-power moderate-duration RF, very-high-power short-duration RF, RF balloon, ultralow-temperature cryoablation, or pulsed field ablation (PFA). Left atrial LGE images were visually assessed and also postprocessed using the full-width half-maximum method for lesion quantification, with gap detection defined by a ≥40% threshold to assess lesion completeness.

**Results:**

Point-by-point RF ablation produced the most continuous LGE lesion patterns, whereas single-shot thermal techniques created broader but less continuous ablation lesions. PFA generated the widest and most heterogeneous patterns, with quantitative analysis confirming a high prevalence and large gap lengths in single-shot ablation groups, most prominently in the PFA group (total LGE encirclement: moderate-power moderate-duration 77.2% ± 10.1%; very-high-power short-duration 82.8% ± 12.4%; RF balloon 76.2% ± 11.6%; ultralow-temperature cryoablation 62.0% ± 9.9%; and PFA 60.2% ± 13.7%).

**Conclusion:**

LGE-CMR reveals modality-specific lesion patterns, with point-by-point RF showing the most continuous PV encirclement. PFA demonstrated variable lesion visualization, likely influenced by system differences and challenges in detecting nonthermal injury. The relationship between CMR-defined lesions and outcomes remains uncertain, underscoring the need for standardized imaging with emerging energy sources.

## Graphical abstract

**Figure:**
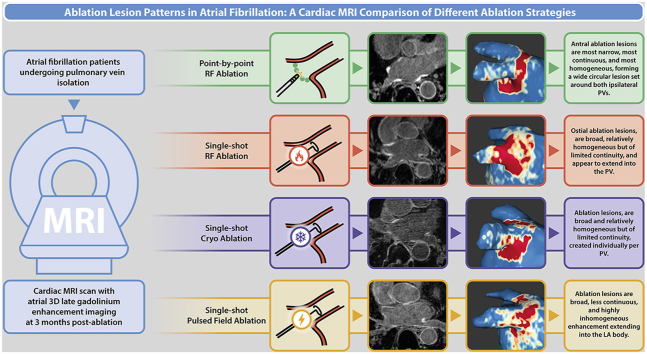

**Key Findings**

▪ Late gadolinium enhancement cardiac magnetic resonance (LGE-CMR) demonstrated ablation modality-specific lesion characteristics, with point-by-point radiofrequency (RF) showing the most continuous and homogeneous pulmonary vein encirclement, whereas cryoballoon and single-shot RF produced broader but less uniform lesions.
▪ PFA produced the widest yet most heterogeneous lesion patterns on LGE-CMR, although reported findings varied across studies, likely reflecting fundamental differences in nonthermal ablation lesion formation and limitations of current imaging and postprocessing methodologies.
▪ The relationship between CMR-defined lesions and clinical outcomes remains unclear, highlighting that standardization of imaging techniques, thresholds, and methodologies, especially with the rise of nonthermal ablation techniques, is crucial to ensure accurate and consistent assessment of lesion durability across modalities.

## Introduction

Over the past decades, catheter ablation for atrial fibrillation (AF) has evolved considerably, shifting from cryoballoon and point-by-point radiofrequency (RF) ablation to a diverse range of technologies designed to enhance safety, efficacy, and procedural efficiency. Among these innovations, single-shot strategies such as balloon-based or circular ablation systems have gained widespread adoption. These approaches provide the potential for more consistent and circumferential pulmonary vein isolation (PVI), with reduced operator dependence and shorter procedure times.^1^, ^2^, ^3^, ^4^ More recently, pulsed field ablation (PFA) has emerged as a promising nonthermal or minimally thermal energy modality that selectively targets myocardial tissue through the mechanism of irreversible electroporation, offering the potential for an even safer and faster ablation strategy.^5^

Despite advances in ablation technologies aimed at improving procedural safety and speed, arrhythmia recurrence remains common, with approximately one-third of patients experiencing AF recurrence within the first year.^1^^,^^5^^,^^6^ Therefore, achieving durable lesion formation is essential for long-term ablation success, yet the persistence and integrity of ablation lesions over time, caused by the different ablation strategies, remain incompletely understood.

Cardiac magnetic resonance (CMR) with late gadolinium enhancement (LGE) imaging is considered a valuable noninvasive surrogate for atrial lesion assessment, reflecting ablation-related scar formation and lesion durability.^7^, ^8^, ^9^, ^10^, ^11^ Distinct LGE patterns have been observed across ablation modalities, likely mirroring differences in tissue injury and healing.^12^, ^13^, ^14^, ^15^ However, systematic comparisons of LGE lesion characteristics between ablation strategies are still limited.

In this study, we evaluated postablation lesion patterns using 3-month post-PVI LGE-CMR across 5 ablation modalities: point-by-point moderate-power moderate-duration (MPMD) RF, very-high-power short-duration (vHPSD) RF, RF balloon, ultralow-temperature cryoablation (ULTC), and PFA.

## Methods

### Study design and study population

The EVALUATE-PVI (NCT05437549, Amsterdam University Medical Center [UMC]), Q-POWER (NCT06846502, Amsterdam UMC), and ENABLE-CMR studies (NCT05432024, St. Antonius Hospital) were prospective, single-center interventional cohort studies conducted in accordance with the Declaration of Helsinki (1964 and its amendments). The study protocols were approved by the local institutional ethics committees (Amsterdam UMC, Amsterdam, and the Medical Research Ethics Committees United). All participants provided a written informed consent.

Patients with paroxysmal or persistent AF referred for a first-time PVI procedure were enrolled if they met Heart Rhythm Society/European Heart Rhythm Association guideline criteria.^16^^,^^17^ Key exclusion criteria included contraindications to CMR (eg, contrast allergy, claustrophobia, incompatible implants or devices, or high body mass index), as well as a history of left atrial (LA) ablation, cardiac surgery, or chest radiation therapy. A CMR was performed 3 months after ablation in all included patients. The current study was designed as an exploratory analysis of available cardiac magnetic resonance imaging (MRI) datasets; therefore, no formal sample size calculation was performed, and no statistical comparisons across studies were conducted.

### Ablation protocol

All PVI procedures were performed under general anesthesia, according to institutional standards and manufacturer instructions. Only PVI was performed; no additional ablation lines were allowed, except cavotricuspid isthmus ablation in the case of documented typical atrial flutter. A detailed ablation protocol is provided in the Supplemental Materials (Supplemental Protocol 1).

#### MPMD point-by-point RF ablation

Ablation was performed using the SmartTouch™ catheter (Biosense Webster, Irvine, CA). Circumferential antral ablation was applied point by point to isolate the ipsilateral pulmonary veins (PVs). Power settings were 40 W for anterior and roof segments and 30 W for posterior and inferior segments, with an inter-tag distance of <6 mm. An ablation index of 550 was targeted for anterior and roof segments, whereas a lower target of 400 was applied to posterior and inferior segments. Touch-up applications were delivered as needed to achieve durable PVI.

#### vHPSD point-by-point RF ablation

Ablation was performed using the QDOT MICRO™ catheter (Biosense Webster) in QMODE+ (90 W). Lesions were delivered in temperature-controlled mode with predefined catheter stability criteria. The inter-tag distance was <4 mm for all segments, except for posterior segments where <6 mm was used. Each application lasted approximately 4 seconds per point. Touch-up applications were delivered in QMODE (<50 W) as needed to achieve durable PVI.

#### Single-shot RF balloon ablation

Using the HELIOSTAR™ RF balloon catheter (Biosense Webster), single-shot RF balloon ablation was performed.^18^ After positioning at the PV antrum, unipolar RF energy (15 W) was delivered for 60 seconds at anterior and 20 seconds at posterior segments. Touch-up ablation was performed if PVI was incomplete.

#### ULTC

ULTC was performed using the Adagio ULTC system (Adagio Medical, Laguna Hills, CA) that enables rapid cooling to the theoretical minimum of −196°C. A flexible stylet-based cryoablation catheter with a circular stylet was used to enable single-shot PVI. Each PV was treated with ≥2 ULTC applications for 60 seconds, followed by an equal thaw time. A 3-dimensional (3D) mapping system guided the ablation. Esophageal protection and phrenic nerve pacing were applied as per protocol, including cryomapping of the right PVs.^19^

#### PFA

The FARAPULSE™ system (Boston Scientific, Menlo Park, CA) was used for PFA. Each PV received 4 ostial (basket) and 4 antral (flower) applications, with approximately 36° catheter rotation after every 2 applications to ensure lesion overlap. Electrical isolation was confirmed using electrograms (entrance and/or exit block) and voltage mapping.

### CMR acquisition protocol

CMR imaging was performed at 3 months after PVI using 1.5T clinical MRI systems equipped with dedicated phased-array cardiac receiver coils. High-resolution, ECG-triggered LGE imaging was obtained using a respiratory-navigated 3D inversion recovery gradient-echo sequence. A gadolinium-based contrast agent (Dotarem, Guerbet, Paris, France) was administered intravenously at a dose of 0.2 mmol per kilogram of body weight, and LGE acquisition started 20 minutes after injection.^20^ The inversion time was individually adjusted to the null signal from healthy myocardium.

At the Amsterdam UMC, imaging was performed on a 1.5T system (Sola, Siemens Healthineers, Erlangen, Germany) with a voxel size of 1.25 × 1.25 × 2.5 mm, reconstructed to 0.625 × 0.625 × 1.25 mm. Typical sequence parameters included a repetition time of 5.2 ms, an echo time of 2.4 ms, and a flip angle of 20°.

At the St. Antonius Hospital, imaging was conducted on a 1.5T system (Ingenia, Philips Healthcare, Best, the Netherlands) with a voxel size of 1.3 × 1.3 × 2.6 mm, reconstructed to 0.65 × 0.65 × 1.3 mm. Sequence parameters included a repetition time of 5.6 ms, an echo time of 2.8 ms, and a flip angle of 15°.

### CMR 3D LGE analysis

3D quantification and visualization of LA LGE was performed using ADAS 3D software (ADAS Medical, Barcelona, Spain). The LA endocardial and epicardial borders were automatically segmented from 3D LGE MRI datasets, with manual corrections applied if necessary after initial interpolation. A 3D reconstruction of the LA was subsequently generated, and non-LA structures, including the PVs, mitral valve, and LA appendage, were excluded from the analysis. Quantification of LGE within the LA body was performed using the full-width at half-maximum (FWHM) technique, classified as border zone + dense fibrosis (LGE >40% of maximal signal intensity), with further subclassification into border zone (40%–60%) and dense fibrosis (>60%). Ablation lesion patterns were visually assessed across ablation strategies, with characterization of lesions (ie, location, extent, distribution, and homogeneity) and gap detection (ie, number and length).

### Statistical analysis

Continuous variables were expressed as mean ± standard deviation for normally distributed data and as median with interquartile range for skewed data. Categorical variables were summarized as frequencies and percentages. Because of the limited sample size, analyses were restricted to descriptive statistics. Analyses were performed using SPSS Statistics, version 28 (IBM Corp, Armonk, NY).

## Results

### Study population

This exploratory study enrolled 42 patients with AF undergoing first-time PVI using 1 of 5 ablation modalities: MPMD (n = 10), vHPSD (n = 10), RF balloon (n = 10), ULTC (n = 4), and PFA (n = 8). Initially, 8 ULTC cases were included; however, 4 were excluded owing to additional posterior wall isolation, compromising PVI lesion assessment (Supplemental Figure 1). Baseline characteristics are presented in Table 1. The mean age was 62 ± 7 years, 78.6% were male, and 59.5% had paroxysmal AF. Postprocedural MRI was acquired 94 days (92–100) after the ablation. Differences in baseline characteristics among the 5 ablation technique groups are presented in Supplemental Table 1.

**Table 1:** Baseline characteristics

Demographics	N = 42
Age, y	62 ± 7
Men	33 (78.6)
BMI, kg/m^2^	25.7 ± 4.1
CHA_2_DS_2_-VASc score of ≥2	10 (23.8)
AF type	
Paroxysmal AF	25 (59.5)
Persistent AF	17 (40.5)
AF duration (mo)	26.5 (14.8–78.8)
Medical history	
Hypertension	7 (16.7)
Diabetes mellitus	1 (2.4)
Coronary artery disease	3 (7.1)
CVA/TIA	5 (11.9)
Sleep apnea	7 (16.7)

Data are expressed as mean ± SD, median (interquartile range), or number (percentage).

AF = atrial fibrillation; BMI = body mass index; CVA = cerebral vascular accident; SD = standard deviation; TIA = transient ischemic attack.

### LA fibrosis burden after ablation

Among the overall cohort (n = 42), the mean percentage of LA body LGE exceeding the 40% signal intensity threshold was 43.6% ± 18.2%. When stratified by LGE burden, the mean proportion classified as border zone fibrosis (signal intensity 40%–60%) was 31.8% ± 16.7%, whereas dense scarring (signal intensity >60%) comprised 11.8% ± 5.0%. Median LA body LGE values according to ablation technique are presented in Table 2.

**Table 2:** 3D LA LGE burden and volumes at 3 months after PVI across ablation modalities

3D LA body	All (N = 42)	MPMD (n = 10)	vHPSD (n = 10)	RF balloon (n = 10)	ULTC (n = 4)	PFA (n = 8)
LGE >40%, (%)	43.6 ± 18.2	46.6 (42.9–56.9)	26.0 (22.9–42.3)	40.4 (27.8–54.8)	27.3 (23.2–42.1)	60.1 (48.4–74.3)
LGE >40% to <60%, (%)	31.8 ± 16.7	33.4 (24.2–43.2)	15.1 (10.1–29.1)	28.4 (18.2–46.8)	20.2 (15.6–28.5)	52.4 (35.9–60.27)
LGE >60%, (%)	11.8 ± 5.0	13.2 (8.2–19.7)	12.8 (9.1–13.7)	11.1 (7.1–12.5)	8.3 (5.2–14.9)	12.1 (6.9–14.5)
Indexed volume, mL/m^2^	46.0 ± 11.6	37.7 (28.6–50.1)	40.4 (34.4–55.9)	42.5 (36.3–48.0)	61.9 (53.8–68.0)	49.1 (45.4–56.2)

Quantification of LA body LGE was performed using the FWHM technique, classified as border zone + dense fibrosis (LGE >40% of maximal signal intensity), with further subclassification into border zone (40%–60%) and dense fibrosis (>60%). 3D LA body volume was indexed to body surface area to compute the indexed 3D LA body volume. Data are expressed as mean ± SD or median (interquartile range).

3D = 3-dimensional; FWHM = full-width at half-maximum; LA = left atrial; LGE = late gadolinium enhancement; MPMD = moderate-power moderate-duration; PFA = pulsed field ablation; PVI = pulmonary vein isolation; RF = radiofrequency; SD = standard deviation; ULTC = ultralow-temperature cryoablation; vHPSD = very-high-power short-duration.

### Ablation lesion patterns

**Figure 1:**
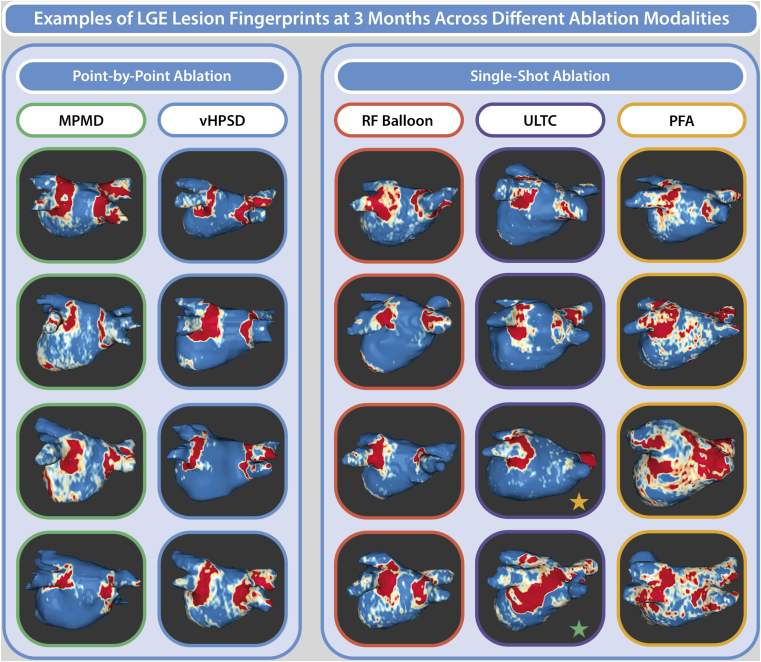
LGE lesion fingerprints 3 months after PVI across different ablation modalities. Point-by-point RF resulted in the most continuous and homogeneous PV encirclement, whereas RF balloon and ULTC ablation produced broader but less uniform lesions. In ULTC case 3 (indicated by the *yellow star*), ablation in the RSPV was discontinued owing to loss of phrenic nerve capture, and no further applications were delivered in the RIPV. Consistent with this, no LGE was observed around the right PVs, illustrating the correspondence between ablation delivery and lesion visualization in this case. In ULTC case 4 (indicated by the *green star*), LGE was observed on the posterior wall despite PVI-only ablation, which may correspond to the low-voltage signals recorded during the procedure and potentially reflect underlying native fibrosis. PFA resulted in detectable lesion formation around the PVs, although it showed the most heterogeneous lesion patterns on CMR. CMR = cardiac magnetic resonance; LGE = late gadolinium enhancement; MPMD = moderate-power moderate-duration; PFA = pulsed field ablation; PV = pulmonary vein; PVI = pulmonary vein isolation; RF = radiofrequency; RIPV = right inferior pulmonary vein; RSPV = right superior pulmonary vein; ULTC = ultralow-temperature cryoablation; vHPSD = very-high-power short-duration.

Ablation lesion patterns varied distinctly across the 5 ablation strategies (Figure 1). Both MPMD and vHPSD point-by-point RF ablation yielded continuous antral lesions, with homogeneous coverage around the ipsilateral veins. Single-shot RF balloon ablation produced broad, homogeneous lesions predominantly located in the ostial PV region, although lesion continuity around the PVs was often limited, especially in the left and right PV roof segments. ULTC resulted in wide and homogeneous antral lesions with inconsistent lesion continuity, specifically on the posterior carina segments. When comparing thermal ablation techniques, 3D LGE-CMR revealed that point-by-point RF ablation produced the most homogeneous and continuous lesion sets encircling the PVs. In contrast, lesion borders after both single-shot ULTC and RF balloon ablation appeared less sharply demarcated, with broader and less homogeneous enhancement patterns. PFA resulted in heterogeneous enhancement patterns, with broad ostial and periantral lesion areas extending into the LA body.

**Figure 2:**
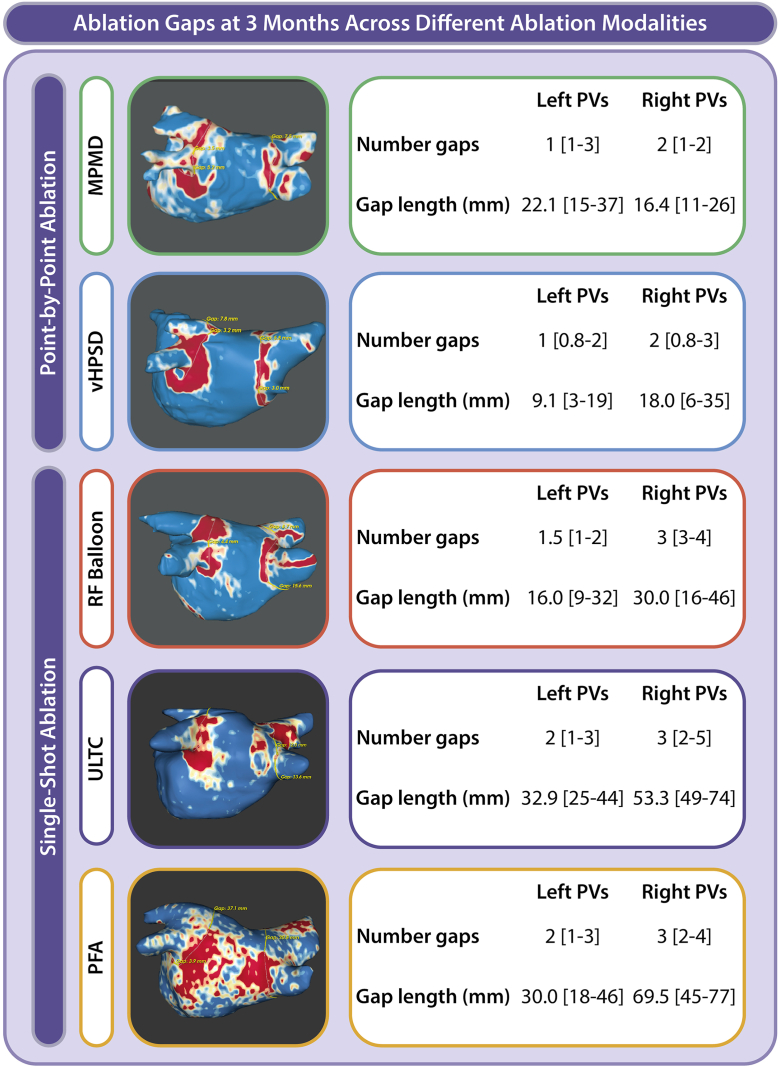
Ablation gap detection on LGE-CMR 3 months after PVI across different ablation modalities. Ablation gap detection on 3D LA LGE models 3 months after PVI across different ablation modalities, applying an FWHM threshold of <60%. Data are expressed as mean ± SD or median (interquartile range). 3D = 3-dimensional; FWHM = full-width at half-maximum; LA = left atrial; LGE-CMR = late gadolinium enhancement cardiac magnetic resonance; MPMD = moderate-power moderate-duration; PFA = pulsed field ablation; PV = pulmonary vein; PVI = pulmonary vein isolation; RF = radiofrequency; SD = standard deviation; ULTC = ultralow-temperature cryoablation; vHPSD = very-high-power short-duration.

These visual observations regarding ablation patterns were further supported by a high prevalence and large extent of gap formation in the thermal single-shot ablation groups, with the most pronounced in the PFA group (total LGE encirclement: MPMD 77.2% ± 10.1%; vHPSD 82.8% ± 12.4%; RF balloon 76.2% ± 11.6%; ULTC 62.0% ± 9.9%; and PFA 60.2% ± 13.7%) (Figure 2, Supplemental Table 2).

## Discussion

This exploratory study provides insights into 3D LGE-CMR–derived ablation lesion characteristics across different ablation strategies for PVI (ie, MPMD, vHPSD, RF balloon, ULTC, and PFA). Despite the modest sample size, distinct and modality-specific ablation lesion patterns were observed, aligning with gap characteristics, consistent with findings from previous single-modality studies; to place these findings in context, an extensive literature search was performed (Supplemental Table 3).

We found that point-by-point RF techniques (MPMD and vHPSD) were associated with more defined and continuous wide antral circumferential lines encircling the PVs. In contrast, single-shot thermal ablation techniques (ie, RF balloon and ULTC) produced broader and less continuous lesion sets localized at the PV antra and frequently induced LGE in the carinal regions. Although our findings align with reports of heterogeneous PFA lesion appearance on LGE-CMR, contrasting studies have shown more consistent and well-demarcated PV encirclement, highlighting the variability in lesion formation and imaging methodology after different PFA methods across different cohorts.

### Point-by-point vs single-shot thermal lesion formation

RF ablation remains 1 of the most widely used energy sources for PVI in the treatment of AF, achieving myocardial injury through a combination of resistive and conductive heating that leads to irreversible necrosis and eventually ablation scar.^21^, ^22^, ^23^ Conventional point-by-point RF ablation is typically performed using MPMD settings (ie, 30–40 W for 20–30 seconds) and primarily creates ablation lesions by using prolonged duration of conductive heat transfer, allowing for deep thermal spread into the myocardium.^22^ In contrast, vHPSD point-by-point RF ablation (90 W for 4 seconds) is designed to confine energy delivery to the resistive phase, creating broader but shallower lesions while limiting collateral injury.^24^, ^25^, ^26^

In the present study, visual assessment of 3D LGE-CMR performed 3 months after PVI demonstrated that lesions created using MPMD point-by-point RF were continuous, forming circumferential lines around both PV pairs. vHPSD lesions were similarly circumferential, but appeared not consistently broader than those created with conventional power settings, despite previous literature suggesting that vHPSD ablation typically produces wider lesion profiles owing to enhanced resistive heating.^27^, ^28^, ^29^ To date, only 1 other study has compared ablation lesion patterns on LGE-CMR between MPMD and vHPSD ablation, reporting a higher percentage of circumferentially encircled PVs in the vHPSD group than conventional settings (40% vs 26%, respectively).^29^ Similarly, our visual assessment, although constrained by the small sample size, indicated more continuous enhancement with vHPSD, accompanied by a relatively low number and short extent of gaps, potentially reflecting greater catheter stability and more efficient energy delivery achieved through brief (and therefore more likely to be stable) tissue contact at high power.^25^ Notably, PVI procedures using vHPSD ablation have been associated with lower AF recurrence and shorter procedure times than MPMD point-by-point RF approaches.^30^^,^^31^ However, the relation between CMR-detected lesion gaps and arrhythmia recurrence remains unclear and should be investigated in future studies.

Although different point-by-point RF ablation strategies have demonstrated comparable durable efficacy for PVI over the years, the use of these techniques is often limited by procedural complexity and variability in individual lesion quality owing to fluctuations in contact force and catheter stability during energy delivery.^32^^,^^33^ Therefore, single-shot ablation techniques have been developed to reduce procedure time and operator dependence by enabling simultaneous circumferential isolation of individual PVs. The RF balloon ablation technique, which delivers thermal energy at a low power and long duration (15 W for 60 seconds), aims to achieve consistent antral contact and generate a uniform circumferential lesion.^4^ On 3D LGE-CMR, enhancement was often diffuse and primarily localized in the PV ostia, consistent with balloon positioning and energy delivery. However, compared with point-by-point techniques, these lesions appeared less continuous and lacked a uniform circumferential encirclement.

Similar findings were observed in the 3D LGE-CMR cases after cryoballoon and ULTC ablation. These thermal ablation modalities produced broad lesions that were more ostially distributed than those from point-by-point ablation, with incomplete circumferential continuity and distinct enhancement along the carina region, an area not routinely ablated in circumferential point-by-point cases.^34^ These observations align with previous reports demonstrating that cryoballoon ablation produces the broadest lesion sets among current modalities.^29^^,^^35^^,^^36^ For example, Regany-Closa et al^29^ demonstrated broader lesion width after cryoballoon vs point-by-point RF ablation (13.3 mm vs 8.7 mm; *P* < .0001). Although cryoablation has been shown to produce a greater overall scar burden, studies also demonstrate a higher prevalence of residual conduction gaps.^12^^,^^29^^,^^35^^,^^37^^,^^38^ LGE-CMR studies by Halbfass et al^39^ and Regany et al^12^ reported PVI encirclement rates of only 33% and 24%, respectively, at 3 months after cryoballoon ablation, substantially lower than the rates observed after point-by-point RF ablation. These observations were consistent with findings from our gap analysis, in which thermal single-shot approaches tended to exhibit a greater number and larger extent of gaps than point-by-point modalities. Although single-shot thermal techniques may offer procedural efficiency, their effectiveness may remain dependent on stable and uniform tissue contact. Therefore, the observed incomplete circumferential continuity, compared with point-by-point ablation, may also reflect differences in delivery systems and catheter design rather than intrinsic properties of the ablation energy source; however, these observations should be interpreted cautiously given the limited sample size and absence of formal statistical testing. Large, prospective studies are required to clarify the relationship between catheter stability, catheter design, tissue contact, and the presence and extent of LGE-defined lesions.^40^

### Thermal ablation vs PFA

Thermal ablation techniques, such as RF and cryoablation, cause tissue injury via temperature-dependent mechanisms. RF ablation creates lesions by achieving cytotoxic temperatures (>50°C) through both resistive and conductive heating, whereas cryoablation induces tissue injury through ice crystal formation, osmotic shifts, and vascular damage during freeze-thaw cycles.^21^^,^^41^, ^42^, ^43^ Both create well-demarcated fibrotic lesions, with dense scar visible on LGE-CMR at 3 months. In contrast, PFA uses nonthermal, high-voltage electric pulses to induce irreversible electroporation, selectively ablating cardiomyocytes, thereby preserving tissue architecture and sparing surrounding structures.^44^^,^^45^

These fundamental differences may also affect scar formation and its visibility on 3D LGE-CMR. Although thermal ablation typically results in robust enhancement at 3 months, findings after PFA are more variable. The study by Regany-Closa et al^29^ was the first to demonstrate distinct ablation lesion patterns across ablation modalities, including PFA, using LGE-CMR at 3 months, showing the lowest proportion of PV encirclement and the largest gap lengths after PFA. In our study, although the findings are interpreted with caution owing to the small sample size and exploratory design, we observed similar modality-specific lesion patterns. These results provide complementary evidence supporting and reinforcing the observations reported by Regany-Closa et al,^29^ highlighting the reproducibility of modality-specific lesion patterns on cardiac MRI. Previous research by Nakatani et al^14^ showed well-demarcated broad lesions in the acute phase after PFA (<3 hours), but a marked reduction in LGE at 3 months, raising questions about the nature of the chronic tissue response. These findings indicate that, besides preserved tissue architecture, PFA lesion evolution may follow a distinct healing trajectory characterized by apoptosis-driven remodeling, limited collagen deposition, and potentially noncollagenous extracellular changes.^46^ As a result, the conventional “wash-in, wash-out” principles underlying LGE may not fully apply, and therefore, PFA-induced injury may be less readily detected with LGE imaging. In the present study, PFA resulted in detectable lesion formation around the PVs, although the enhancement appeared limited, continuous, and very heterogeneous, corresponding with the detection of relatively large gaps. Conversely, 2 studies using different PFA systems (FARAPULSE and VARIPULSE) demonstrated high rates of bilateral PV encirclement (Sohns et al^13^ 92.7% vs Fink et al^15^ 80%) on LGE-CMR at 3 months after PVI. The variability in PV enhancement at 3 months may reflect not only fundamental biological differences in lesion formation but also influences of the PFA system, pulse protocol, and catheter design.^46^, ^47^, ^48^ The present study, like most to date, evaluated PFA exclusively with the FARAPULSE system, whereas emerging evidence indicates that different PFA platforms (FARAPULSE, VARIPULSE, PulseSelect) may generate distinct lesion patterns.^49^ Moreover, previous reports suggest that PFA may not be purely nonthermal, with variable degrees of thermal contribution that could further shape lesion heterogeneity and LGE signal characteristics, dependent on catheter design and delivery parameters, many of which remain proprietary.^47^^,^^49^, ^50^, ^51^, ^52^ Despite variable findings regarding PFA, recent pooled remapping data from 22 patient cohorts demonstrated superior per-vein durability with PFA compared with thermal ablation, emphasizing that lesion interpretations based on thermal ablation may not accurately capture PFA lesion formation on LGE-CMR.^53^ Further investigation into the histopathologic correlates of LGE after different PFA systems is warranted, given that current PFA lesion assessment may be influenced by multiple factors, including electrophysiological variables (ie, system, catheter design and pulse train) and imaging factors (ie, contrast-agent kinetics, timing of acquisition, technique, and postprocessing).

### Role of 3D LGE-CMR lesion assessment

LGE-CMR has emerged as a valuable modality for the noninvasive evaluation of atrial ablation lesions.^9^^,^^11^^,^^37^^,^^54^^,^^55^ Nonetheless, interpretation and quantification of atrial enhancement remain technically challenging, primarily owing to the thin atrial wall, limited spatial resolution of CMR, and cardiac motion artifacts. Moreover, the literature to date reflects substantial methodological heterogeneity: patients were imaged on different MRI platforms with different field strengths, using diverse image acquisition protocols, postprocessing software, and quantification strategies (eg, Utah staging, image intensity ratio, or FWHM approaches).^20^^,^^54^^,^^56^ Results have been reported in disparate units (percent scar burden, milliliters of enhancement, or square centimeters of lesion area), and the extent of LGE quantification has varied, with some studies measuring only peri-PV LGE and others assessing the entire LA body. These factors complicate direct comparison between studies and limit robust synthesis of the available evidence.

This means that certain ablation strategies may appear less continuous or less extensive on LGE, not owing to insufficient ablation but because they may fall below the relative enhancement threshold. Consequently, lesion width and gap detection are highly threshold and scar quantification method dependent, with no standardized MRI-based definition of conduction gaps. For PFA in particular, the biological meaning of LGE remains uncertain, given that its nonthermal mechanism may lead to cell death pathways with limited collagen deposition and altered extracellular remodeling not fully captured by conventional atrial LGE assessments. Recently, however, Hermans et al^57^ reported that PFA lesions may be quantified using thresholds similar to those applied for thermal ablation; however, confirmation in larger, multicenter cohorts is still needed.

Despite these technical limitations and the variability inherent to previous studies, both the existing literature and the present investigation consistently demonstrate that, especially in RF point-by-point ablation, LGE-CMR reveals circumferential lesions surrounding the PVs.^58^, ^59^, ^60^, ^61^, ^62^, ^63^, ^64^, ^65^, ^66^, ^67^, ^68^, ^69^, ^70^, ^71^, ^72^, ^73^, ^74^ Standardization of imaging protocols, quantification strategies, and reporting metrics will be essential to enable more reliable interstudy comparisons and to define the relationship between CMR-defined lesions and long-term clinical outcomes. In this context and in the absence of systematic correlation with arrhythmia recurrence or invasive remapping data, heterogeneous or less continuous LGE, particularly after PFA, should not be assumed to represent inferior lesion durability or interpreted as a surrogate for clinical efficacy. However, establishing this link is critical to determine the clinical utility of LGE as a marker of lesion durability and arrhythmia recurrence risk, ultimately guiding procedural refinement and decisions regarding repeat intervention. Accordingly, future studies integrating LGE-CMR with invasive electroanatomic remapping will be essential to determine the relationship between imaging-defined gaps and functional gaps, particularly for emerging PFA techniques, in which the relationship between chronic LGE characteristics and durable isolation remains incompletely understood.

### Limitations

This was an exploratory, descriptive study with a relatively small sample size, which precluded statistical analysis and may limit the study’s generalizability. This also restricted comparison of CMR-defined lesion characteristics between ablation techniques and their association with long-term procedural durability and clinical outcomes. Furthermore, the EVALUATE-PVI/Q-POWER and ENABLE-CMR studies were conducted across 2 centers using different MRI scanners (Siemens and Philips), which may have introduced variability in image quality and lesion visualization between modalities. In addition, the study population included both patients with paroxysmal and persistent AF, with a higher proportion of persistent AF in the PFA group; thus, a more extensive atrial myopathy associated with persistent AF may have contributed to the observed LA LGE at 3 months. Moreover, the LGE signal may in part reflect native fibrosis, atrial myopathy, or extracellular expansion from edema or inflammation, rather than ablation injury alone. Absolute LGE lesion width and gap detection may vary with different imaging methodologies and quantification thresholds, differences that are widely reflected in the literature and may yield divergent results, thereby complicating direct comparisons. Defining the most appropriate approach for thermal and nonthermal lesions will require future studies incorporating standardized imaging workflows, larger cohorts, invasive remapping, and clinical outcome data.

## Conclusion

This exploratory study used visual and quantitative assessment of 3D LGE-CMR to characterize lesion patterns across different ablation modalities for PVI. In line with previous studies, we observed modality-specific differences in lesion appearance: point-by-point RF produced the most continuous circumferential enhancement, whereas single-shot thermal approaches yielded broader but less uniform lesions. PFA demonstrated more heterogeneous signal characteristics. Although these findings should be interpreted cautiously given the exploratory design and limited sample size, they provide complementary imaging-based evidence supporting previously reported modality-specific lesion patterns. These observations also highlight the influence of energy source and postprocessing methodology on lesion visualization and underscore the need for further studies and standardized CMR approaches to better characterize ablation lesions, particularly with the emergence of nonthermal ablation technologies.

## Disclosures

The authors have no conflicts of interest to disclose.

## Figures and Tables

**Supplementary Figure:**