# Supplementary material for: Ablation lesion patterns in atrial fibrillation: A cardiac MRI comparison of different ablation strategies
Source: Heart Rhythm O2. 2026 Apr 8;7(7):1304–13. doi: 10.1016/j.hroo.2026.03.035 (PMC13390004; doi:10.1016/j.hroo.2026.03.035)
Supplement: Supplementary Material [file mmc1.pdf]

Supplemental Material files

Protocol S1. Ablation strategies

Point-by-point and single-shot RF

The RF ablation procedures (EVALUATE-PVI and Q-POWER Study) were performed in the Amsterdam UMC under general anesthesia. Circumferential point-by- point antral ablation was performed using the SmartTouch^{TM} or QDOT MICRO^{TM} ablation catheter (J&J MedTech Electrophysiology) to create continuous lesions isolating ipsilateral PVs. Ablation settings for the SmartTouch were: 40 W and ablation index target of 550 for anterior/roof segments, and at 30/35 W and ablation index target of 400 for posterior/inferior segments; and QDOT: QMODE+ (90 W) with an inter tag distance (ITD) of <4 mm for all segments, except for the posterior superior and posterior inferior segments where the ITD of <6 mm was kept. Touch-up RF applications after initial encircling of the PVs were performed if required to achieve PVI. Single-shot ablation was performed using the HELIOSTAR^{TM} RF balloon catheter (J&J MedTech Electrophysiology). After positioning the RF balloon at the antra of each PV, the balloon was inflated and irrigated at a rate of 35 ml/min. When optimal contact of all 10 electrodes was achieved, ablation energy was delivered in unipolar mode at 15 W, maintaining a target temperature of 55°C for 60 seconds at the anterior segments and 20 seconds for posterior segments. Touch-up RF ablation was performed by another circumferential or segmental ablation if required to achieve PVI.

After a waiting period of 30 min, durability of PVI was confirmed by bidirectional conduction block. If acute reconnection occurred, further ablation was performed until re-isolation was achieved, followed by a new waiting period of 30 minutes. Additional ablation lines were not

allowed, with the exception of cavotricuspid ablation in case of documented typical counterclockwise atrial flutter.

Ultra-low temperature cryo

The ULTC ablation procedures (Adagio Medical Inc., Laguna Hills, CA, USA) were performed in the St. Antonius hospital under general anesthesia. A 3D-mapping system (EnSite X, Abbott, Abbott Park, IL, USA) was used during the procedure to guide the ablation. A warming balloon was positioned in the esophagus prior to ablation. The flexible ULTC ablation catheter was positioned in the left atrium and configured to a circular shape using the appropriate stylet. The ULTC system enabled rapid cooling of the ablation catheter to theoretical minima of -196 degrees Celsius. Ablation of the PVs included at least 2 applications per vein for a duration of 60 seconds, followed by an equal thaw time. The catheter was repositioned after each application. Ablation of the right PVs was performed under continuous phrenic nerve pacing and “cryomapping” (cryoablation using temperatures above the ultra-low range) to monitor for phrenic nerve paresis.

Pulsed field ablation

The pulsed field ablation procedures were performed in the St. Antonius hospital under general anesthesia with the use of the FARAPULSE (Boston Scientific, Marlborough, MA, USA) system. This system delivers applications consisting of bipolar, biphasic electrical pulse trains with a voltage output of 2.0kV. After collecting baseline 3D-mapping voltage images with ENSITE X, each PV was treated with 4 ostial (basket) and 4 antral (flower) applications. After every 2 applications, the catheter was rotated approximately 36° to ensure overlapping lesions. Electrical isolation was confirmed by assessing for entrance- and/or exit- block using

the circular mapping catheter and additional voltage mapping. In case of incomplete isolation of a pulmonary vein, additional applications were delivered.

Figure S1. Examples of excluded ULTC cases with PVI + posterior wall isolation

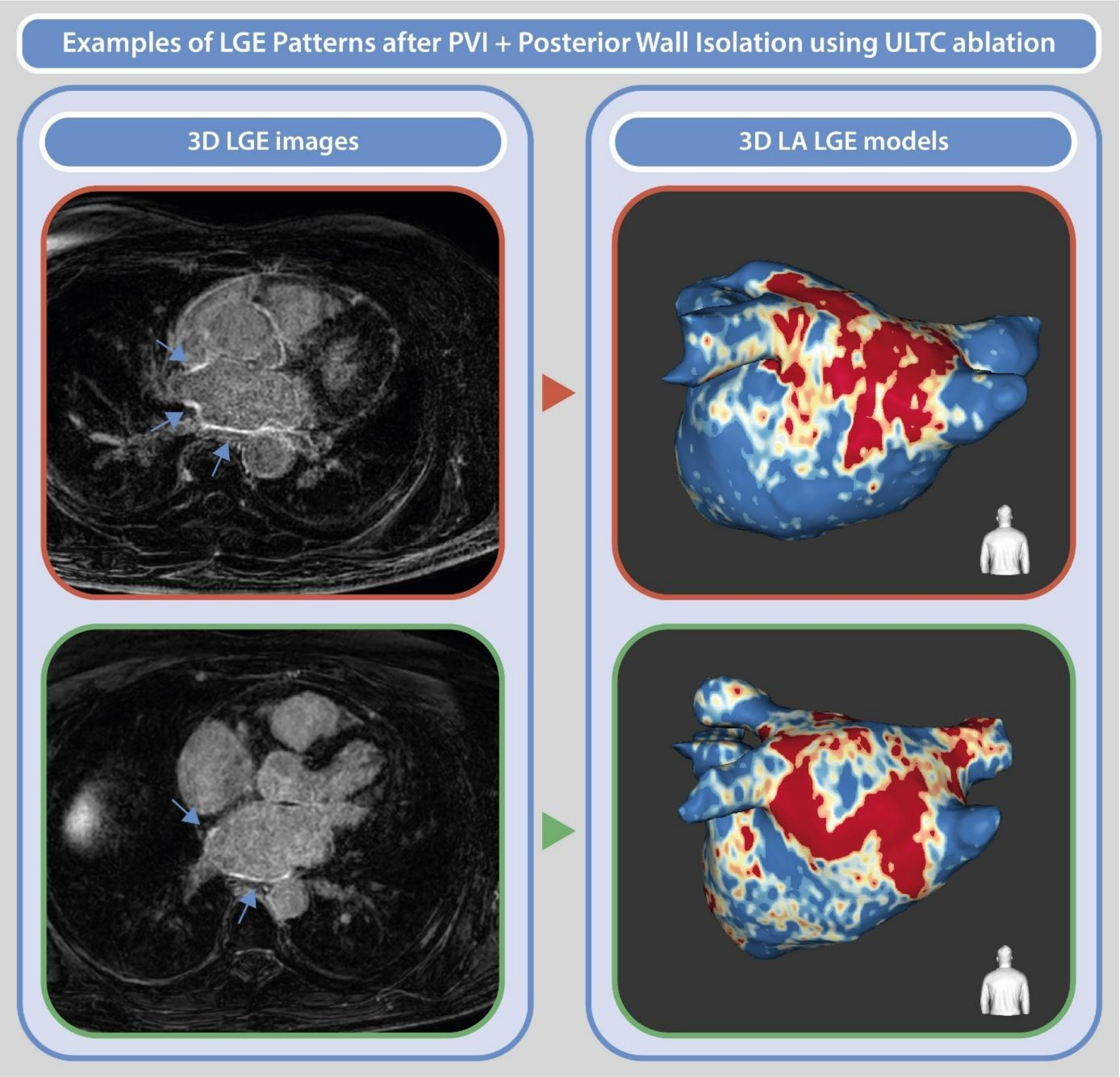

Two examples of patients excluded from the main analysis due to additional posterior wall isolation, which compromised true PVI lesion assessment. Blue arrows on the 3D LGE images indicate areas of high signal intensity, displayed in red on the 3D LA LGE models in ADAS, corresponding to ablation of the posterior wall.

Table S1. Baseline characteristics across different ablation modalities

Demographics	*All* *N=42*	*MPMD* *N=10*	*vHPSD* *n=10*	*RF balloon* *N=10*	*ULTC* *N=4*	*PFA* *N=8*
**Age, years**	61.6 ± 6.6	60.8 ± 8.9	61.1 ± 4.6	61.0 ± 4.3	67.0 ± 9.0	61.5 ± 6.8
**Male, n (%)**	33 (78.6%)	7 (70%)	9 (90%)	7 (70%)	3 (75%)	7 (87.5%)
**BMI, kg/m²**	25.7 ± 4.1	25.2 ± 5.3	27.0 ± 3.5	25.1 ± 4.5	25.7 ± 5.4	25.6 ± 2.4
**CHA_2DS_2-VASc ≥ 2**	10 (23.8%)	2 (20%)	2 (20%)	3 (30%)	2 (50%)	1 (12.5%)
AF type, n (%)						
**Paroxysmal AF**	25 (59.5%)	8 (80%)	5 (50%)	7 (70%)	2 (50%)	3 (37.5%)
**Persistent AF**	17 (40.5%)	2 (20%)	5 (50%)	3 (30%)	2 (50%)	5 (62.5%)
**AF duration (months)**	26.5 [14.8 – 78.8]	53.5 [25 – 106.5]	19.0 [10.8 – 64.5]	74.0 [22.5 – 127.5]	34.5 [17.0 – 79.0]	13.5 [7.3 – 21.5]
Medical history, n (%)						
**Hypertension**	7 (16.7%)	2 (20%)	3 (30%)	1 (10%)	1 (25%)	0 (0%)
**Diabetes Mellitus**	1 (2.4%)	0 (0%)	0 (0%)	0 (0%)	0 (0%)	1 (12.5%)
**Coronary artery disease**	3 (7.1%)	2 (20%)	1 (10%)	0 (0%)	0 (0%)	0 (0%)
**CVA/TIA**	5 (11.9%)	3 (30%)	1 (10%)	1 (10%)	0 (0%)	0 (0%)
**Sleep apnea**	7 (16.7%)	3 (30%)	1 (10%)	0 (0%)	1 (25%)	2 (25%)

Data are expressed as mean ± SD, median (interquartile range), or number (percentage). Abbreviations: reported in previous tables/figures.

Table S2. Ablation gap detection and measurements across different ablation modalities

Gap detection	All<br>N=42	MPMD<br>N=10	vHPSD<br>N=10	RF balloon<br>N=10	ULTC<br>N=4	PFA<br>N=8
**Number of gaps**						
**Left PVs**	1.5 [1-2]	1 [1-3]	1 [0.75-2.0]	1.5 [1-2.3]	2 [1.3-2.8]	2.0 [1.3-2.8]
**Right PV**	2.5 [1.8-3.0]	2 [1-2]	2.0 [0.75-3.0]	3.0 [2.5-4.0]	3.0 [2.0-4.8]	3.0 [2.3-4.0]
**Gap length, mm**						
**Left PVs**	20.5 [10.1-34.3]	21.1 [14.9-36.7]	9.1 [2.8-18.6]	16.0 [9.2-32.1]	32.9 [24.9-43.7]	30.0 [18.0-45.8]
**Right PV**	29.5 [13.4-48.1]	16.4 [10.7-26.0]	18.0 [6.2-35.3]	30.0 [15.7-45.6]	53.3 [48.5-74.3]	69.5 [45.0-77.4]
**LGE encirclement, %**						
**Left PVs**	76.3 ± 15.6	71.3 ± 14.9	84.8 ± 17.6	78.3 ± 14.6	72.2 ± 11.4	71.6 ± 15.5
**Right PV**	70.6 ± 20.8	81.9 ± 13.7	80.9 ± 15.0	74.6 ± 15.0	50.3 ± 14.7	49.0 ± 22.1
**Total PVI**	73.6 ± 14.1	77.2 ± 10.1	82.8 ± 12.4	76.2 ± 11.6	62.0 ± 9.9	60.2 ± 13.7

Gap detection and measurements using 3D LA LGE models 3 months post-PVI using FWHM<60%. Data are expressed as mean ± SD or median [interquartile range]. Abbreviations: in previous tables/figures.

Table S3. Literature review of LGE-CMR outcomes following various ablation modalities for PVI

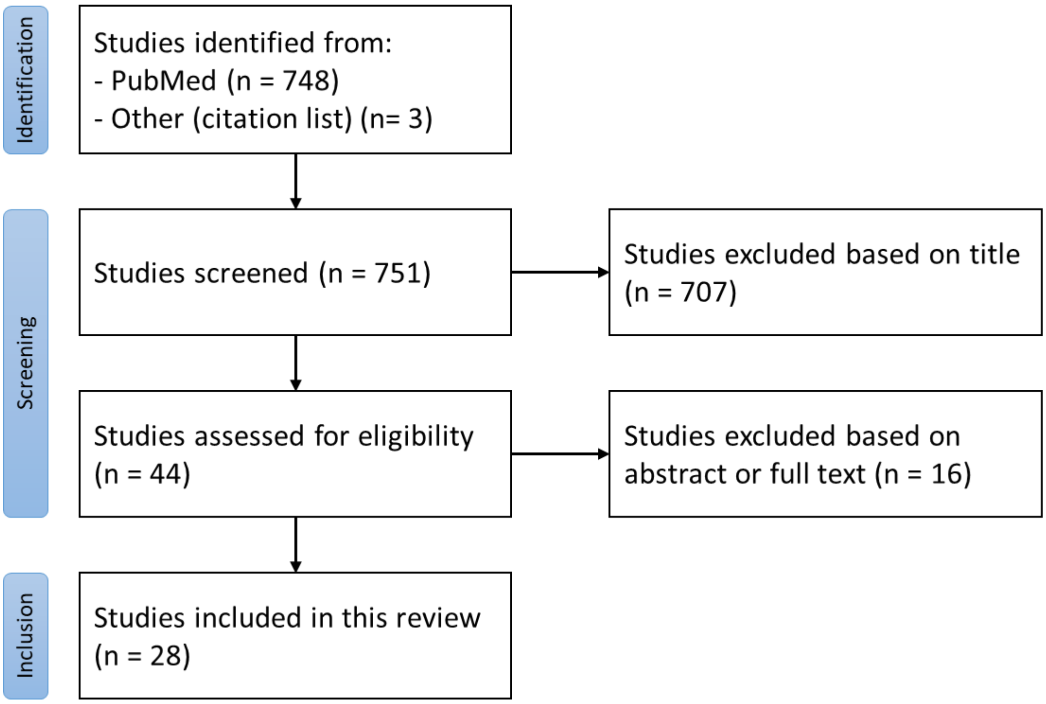

First author	Year	Ablation modality	Scanner	LGE quantification	Most important CMR findings
McGann et al.[7]	2008	RF point-by-point[1] (n= 46)	1.5T[a]	Software: OsiriX & Matlab<br>LGE quantification: voxel intensity of 3 SD above healthy tissue mean pixel intensity.	LA scar burden:<br>- PVI non-responders: 12.4 ± 5.7%<br>- PVI responders: 19.3 ± 6.7%
Wylie et al. [58]	2008	RF point-by-point[1] (n= 33)	1.5T[b]	Software: Mathlab<br>LGE quantification: patient-specific threshold, average 3.6 SD above blood signal.<br>NB: CMR performed 1-2 months post PVI	Mean scar volume: 8.1 ± 3.7 mL
Badger et al.[59]	2010	RF point-by-point[1] (n= 154)	1.5T[a]	Software: Matlab<br>LGE quantification: Voxel intensity 3 SD above healthy tissue mean pixel intensity.<br>NB: PVI + posterior wall + septal debulking	Circumferentially scarring of all PV's: 11.1% of patients
Arujuna et al. [60]	2012	RF point-by-point[1] (n= 172)	1.5T[b]	Software: STATA<br>LGE quantification: Voxel intensity above healthy tissue mean pixel intensity.	LA scar burden:<br>- PVI non-responders: 34 %(95% CI 26.7-41.3%)<br>- PVI responders: 60% (95% CI 54.3-65.7%)

Halbfass et al. [39]	2014	Cryoballoon[2] (n= 30)	3.0T[c]	Software: Corview<br>LGE quantification: normalized voxel intensity.	Ratio of circumferentially encircled PVs: 33%<br>Complete circumferential ablation lesions: 62% of left PVs vs. 7% of right PVs ($p < 0.001$)
Akoum et al. [37]	2015	RF point-by-point[1] (n= 157)<br>Cryoballoon[2] (n= 12)<br>RF single-shot[3] (n= 8)	1.5T and 3.0T[d]	Software: Corview<br>LGE quantification: voxel intensity of 2 SD above healthy tissue mean pixel intensity.<br>NB: 62.7% PVI only, 29.4% additional ablation, 7.9% other.	LA scar burden:<br>- RF: 10.5 ± 4.3%<br>- Cryoballoon: 13.4 ± 6.2%<br>- RF single-shot: 7.1 ± 2.3%<br>Ratio of circumferentially encircled PVs: 7.3%
Akoum et al. [61]	2015	RF point-by-point[1] (n= 172)	3.0T[c]	Software: Corview<br>LGE quantification: normalized voxel intensity.	Circumferentially scarring of all PV's: 8.7% of patients
Khurram et al. [38]	2015	RF point-by-point[1] (n= 7)<br>Cryoballoon[4] (n= 5)	1.5T[a]	Software: OsiriX and QMass MR<br>LGE quantification: the IIR relative to the blood pool, threshold of ≥0.97.	LA scar burden:<br>- RF: 46.3 ± 3.6% (pre-PVI: 29.0 ± 6.8%)<br>- Cryoballoon: 50.0 ± 8.6% (pre-PVI: 38.0 ± 10.7%)
Figueras I Ventura et al.[62]	2018	RF point-by-point (n= 15)	3.0T[d]	Software: ADAS 3D<br>LGE quantification: IIR relative to the blood pool, threshold of ≥1.2.	Ratio of circumferentially encircled PVs: 19.3%.<br>LA scar area compared to baseline CMR: 16.4 [IQR 9.8-27.0] cm^2
Kurose et al. [63]	2018	RF point-by-point[1] (n= 13)<br>Cryoballoon[2] (n= 29)	1.5T[b]	Software: Ziostation<br>LGE quantification: Voxel intensity 2-4 SD above healthy tissue mean pixel intensity.<br>NB: CMR performed 1-3 months post PVI	Mean ablation lesion width:<br>- RF: 5.6 ± 2.0mm<br>- Cryoballoon: 8.2 ± 2.2mm
Linhart et al. [64]	2018	RF dragging strategy[1] (n= 94)	3.0T[d]	Software: ADAS 3D<br>LGE quantification: IIR relative to the blood pool, threshold of ≥1.2.	Circumferentially scarring of all PV's: 4% of patients<br>Average number of gaps per patient: 5.4.<br>Median gap length: 7.3 mm [IQR 4.9-13.0]
Jefairi et al.[65]	2019	RF point-by-point[1] (n= 28)<br>RF single-shot[5] (n= 23)	1.5T[a]	Software: MUSIC<br>LGE quantification: threshold of 50-70% of maximum signal intensity.	Scar burden (mL) and gaps in scar formation (%):<br>- Point-by-point: 7.1 ± 2.2 mL; 86% of patients<br>- Single-shot: 8.6 ± 1.7 mL; 65% of patients
Akita et al. [66]	2019	Cryoballoon[2] (n= 40)<br>Hot-balloon[10] (n= 40)	1.5T[b]	Software: Ziostation<br>LGE quantification: Voxel intensity 2-4 SD above healthy tissue mean pixel intensity.	Mean number of gaps and lesion width:<br>- Cryoballoon: 2.9 ± 2.4; 7.8 ± 2.0 mm<br>- Hot-balloon: 1.3 ± 1.4; 4.9 ± 1.0 mm
O'Neill et al. [67]	2019	RF point-by-point[1] (n= 26)<br>RF dragging strategy[1] (n= 20)	1.5T[e,f]	Software: SPSS & Prism<br>LGE quantification: Voxel intensity 3.3 SD above mean blood pool signal intensity.	Ratio of circumferentially encircled PVs (%), mean scar burden (%) and total scar width (mm):<br>- Point-by-point: 19%; 6.6 ± 6.8%; 7.9 ± 3.6mm<br>- Dragging strategy: 0%; 9.6 ± 5.0%; 10.7 ± 2.3mm
Alarcón et al. [68]	2020	RF dragging strategy[1] (n= 30)<br>Cryoballoon[2] (n= 30)	3.0T[d]	Software: ADAS 3D<br>LGE quantification: IIR relative to the blood pool, threshold of ≥1.2.<br>NB: Propensity score matching	Mean number of gaps and gap length (mm):<br>- RF: 5.2 ± 2.3; 17.4 ± 14.1 mm<br>- Cryoballoon: 4.5 ± 2.1; 18.2 ± 13.0 mm

Kirstein et al. [69]	2020	Cryoballoon[2] (n= 26)	1.5T[a, c]	Software: Corview<br>LGE quantification: normalized voxel intensity.	LA scar burden: 19.4% (absolute increase of 9.3 ± 3.7% compared to baseline CMR)
Kurose et al.[35]	2020	RF point-by-point[6] (n= 12)<br>Cryoballoon[2] (n= 18)	1.5T[b]	Software: Ziostation<br>LGE quantification: voxel intensity of 2 SD above healthy tissue mean pixel intensity.<br>NB: CMR performed 1-3 months	Scar lesion width and gaps in scar formation:<br>- RF: 6.3 ± 2.2mm; gaps 13%<br>- Cryoballoon: 8.1 ± 2.2mm; gaps 22%
Trotta et al. [70]	2020	Cryoballoon (n= 49)	3.0T[e]	Software: ADAS 3D<br>LGE quantification: IIR relative to the blood pool, threshold of ≥1.2.	Average number of gaps per PV: 1.3 ± 1.0<br>Ratio of circumferentially encircled PVs: 24%
Nakatani et al.[14]	2021	PFA[7] (n= 18)<br>RF point-by-point[1] (n= 7)<br>Cryoballoon[2] (n= 16)	1.5T[e]	Software: MUSIC<br>LGE quantification: FWHM, the maximum signal intensity as an internal reference and a threshold set at 50% maximum intensity.	No discrete values of scar at 3 months.<br>Reversibility of 60% for PFA and 18% for thermal PVI as compared to MRI within 3 hours post-PVI.
Nelson et al.[71]	2022	RF point-by-point[1] (n= 313)<br>Cryoballoon[2] (n= 51)	1.5T[d]	Software: Merisight<br>LGE quantification: voxel intensity of 2-4 SD above healthy tissue mean pixel intensity.	LA scar burden:<br>- RF 8.8 ± 4.2%<br>- Cryoballoon 6.4 ± 3.6%
Takahara et al. [72]	2022	RF point-by-point[6] (n= 30)	1.5T[b]	Software: Ziostation & MRI LADE analysis<br>LGE quantification: Voxel intensity 2-4 SD above healthy tissue mean pixel intensity.	Total lesion volume: 6.3 ± 2.6 mL<br>Average number of gaps per PV: 2.5 ± 1.8<br>Average gap length: 7.7 ± 5.3mm
Rav Acha et al. [73]	2023	Cryoballoon[2] (n= 22)	1.5T[e]	Software: ADAS 3D<br>LGE quantification: IIR relative to the blood pool, threshold of ≥1.2.<br>NB: CMR performed 3-6 months post PVI	Ratio PV's with complete or subcomplete (only minor gaps) circumferential fibrosis: 78.8%
Regany et al. [12]	2023	RF point-by-point[1] (n= 47)<br>Cryoballoon[2] (n= 40)	3.0T[g]	Software: ADAS 3D<br>LGE quantification: IIR relative to the blood pool, threshold of >1.2.	Ratio of circumferentially encircled PVs and number of gaps:<br>- RF: 39%, gaps: 2.7 per patient<br>- Cryoballoon: 24%, gaps: 3.2 per patient
Sohns et al. [13]	2023	PFA[7] (n= 10)	N.R.	Software: Merisight<br>LGE quantification: voxel intensity of 2-4 SD above healthy tissue mean pixel intensity.<br>NB: PVI and posterior wall isolation	LA scar burden: 8.1 ± 2.1%<br>Mean scar width: 12.8 ± 2.1mm<br>Bilateral PV encirclement: 37 out of 40 PVs (92.7%)
Sciacca et al. [28]	2023	vHPSD RF point-by-point[8] (n= 30)	3.0T[b]	Software: Merisight<br>LGE quantification: voxel intensity of 2-4 SD above healthy tissue mean pixel intensity.	LA scar burden: 9.5 ± 1.9%<br>Mean scar width: 13.6 ± 2.9mm<br>Gaps in scar formation: 10 patients (33.3%), mean 1.8 ± 1 gap/patient
Watanabe et al. [74]	2023	Cryoballoon[2] (n= 24)<br>Hot-balloon[10] (n= 24)	1.5T[b]	Software: ADAS-AF<br>LGE quantification: IIR relative to the blood pool, threshold of ≥1.2.<br>Threshold of ≥1.32 for chronic ablation lesion area	Mean scar lesion area (=chronic lesion)<br>- Cryoballoon: 34.1 ± 15.6 cm^2<br>- Hot balloon: 29.4 ± 15.9 cm^2

				NB: Propensity score matching	
Fink et al. [15]	2025	PFA[9] (n= 20)	N.R.	Software: Merisight<br>LGE quantification: voxel intensity of 2-4 SD above normal/healthy tissue mean pixel intensity.	Circumferential scarring of all PVs: 16 patients (80%)<br>LA scar burden: 6.1 ± 1.9%<br>Mean scar width: left PVs 12.1 ± 3.1mm; right PVs: 10.7 ± 2.3mm.
Regany-Closa et al. [29]	2025	RF point-by-point[1] (n= 43)<br>vHPSD RF point-by-point[8] (n= 25)<br>Cryoballoon[2] (n= 40)<br>PFA[7] (n= 30)	3.0T[g]	Software: ADAS 3D<br>LGE quantification: IIR relative to the blood pool, threshold of ≥1.2.	Ratio of circumferentially encircled PVs (%) and mean lesion width (mm):<br>- RF: 26%, 8.7 mm<br>- vHPSD: 40%, 10.9 mm<br>- Cryoballoon: 24%, 13.3 mm<br>- PFA: 12%, 12.7 mm

Abbreviations: CMR, cardiac magnetic resonance; FWHM, full-width half-max; IIR, image intensity ratio; IQR, interquartile range; N.R., not reported; PV, pulmonary vein; PVI, pulmonary vein isolation; others as reported in previous tables.

Ablation modality
[1]THERMOCOOL SMARTTOUCH/NAVISTAR, Biosense Webster
[2] ArcticFront (Advance), Medtronic
[3]PVAC, Medtronic
[4]Freezor MAX, Medtronic
[5]nMARQ, Biosense Webster
[6]TactiCath, Abbott
[7]FARAPULSE, Boston Scientific
[8]QDOT Micro, Biosense Webster
[9]VLC, Varipulse, Biosense Webster
[10]SATAKE Hot-balloon, Toray industries

Scanner
[a]AVANTO, Siemens
[b]Achieva, Philips Medical
[c]Verio, Siemens
[d]Trio-Tim, Siemens
[e]AERA, Siemens
[f]Ingenia, Philips
[g]PRISMA, Siemens
